# Anomalous Right Coronary Artery With Interarterial Course Presenting As Exercise-Induced Inferior ST-Elevation Myocardial Infarction

**DOI:** 10.7759/cureus.109696

**Published:** 2026-05-26

**Authors:** Shan Zaidi, Ashley M Porter, Satyajeet Roy

**Affiliations:** 1 Department of Cardiology, Cooper Medical School of Rowan University, Camden, USA; 2 Department of Cardiology, Cooper University Hospital, Camden, USA; 3 Department of Medicine, Cooper Medical School of Rowan University, Camden, USA

**Keywords:** anomalous right coronary artery, coronary artery bypass grafting (cabg), interarterial course, multimodality imaging, st-elevation myocardial infarction

## Abstract

An anomalous right coronary artery (ARCA) is a congenital coronary artery condition in which the right coronary artery (RCA) originates from an abnormal position. The most common locations of an ARCA are the left sinus of the Valsalva (LSV) and the left main coronary artery (LMCA). In this case, a 49-year-old male with hyperlipidemia presented with chest pain radiating to both arms and back after a high-intensity exercise program. Electrocardiogram demonstrated an inferior wall ST-elevation myocardial infarction (STEMI). The patient received thrombolytic therapy and underwent percutaneous coronary intervention on the mid left circumflex artery with initiation of dual antiplatelet therapy. Coronary computed tomography angiography revealed an ARCA origin. The patient was referred to cardiothoracic surgery and subsequently underwent successful coronary artery bypass grafting (CABG) with discharge in stable condition. ARCA is a rare congenital condition that should be considered in patients with myocardial ischemia or sudden cardiac death. Compression of the RCA between the aorta and pulmonary artery in an interarterial course may cause exertional ischemia and lead to sudden death. Furthermore, ARCA is associated with a stenotic ostium and an acute angle of origin. Management options include: Reimplantation of the RCA, CABG, and unroofing of the intramural segment. Our case emphasizes awareness of ARCA in clinical practice and prompt management.

## Introduction

An anomalous right coronary artery (ARCA) is a well-recognized congenital coronary anomaly; however, the specific variant involving the right coronary artery (RCA) arising from the left coronary sinus with an interarterial course is relatively uncommon, with a reported prevalence of 0.23-0.26% [1-2]. In this variant, the RCA traverses between the aorta and pulmonary artery, which is often associated with an intramural segment within the aortic wall. This intramural segment is now thought to play a larger role in myocardial ischemia than simply compression between the great vessels [3-4]. Notably, high-risk features associated with ARCA are slit-like ostium, acute takeoff angle, and proximal luminal narrowing. These features limit coronary blood flow, especially during periods of increased cardiac demand such as exercise [3-5]. In patients with ARCA, sudden cardiac death has most often been reported during or shortly after exertion, and autopsy studies frequently have shown evidence of chronic subclinical ischemia [4-6]. Commonly, patients present with syncope, exertional chest pain, or arrhythmias, and thus presentation as an acute ST-elevation myocardial infarction (STEMI) is exceedingly rare [6-7]. We present a case of an ARCA with an interarterial course presenting as an acute STEMI following intense exertion, ultimately requiring surgical correction.

## Case presentation

**Figure 1:**
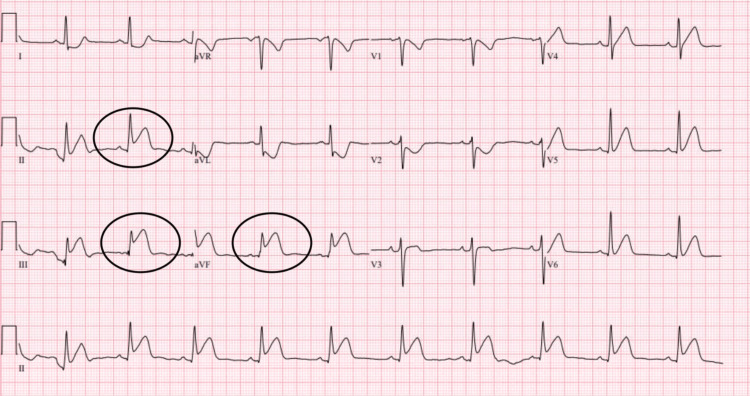
Electrocardiogram ST-segment elevation in the inferior leads (II, III, and aVF), highlighted by circles and consistent with an inferior myocardial infarction.

A 49-year-old man with hypothyroidism and hyperlipidemia presented to an outside hospital with acute substernal chest pain radiating to the left chest and was found to have an inferior STEMI on electrocardiogram (Figure 1). Symptoms began shortly after participating in a high-intensity exercise program earlier that day. On arrival to the emergency department, vital signs were notable for blood pressure of 172/101 mmHg, pulse 65 beats/min, respiratory rate 20 breaths/min, and oxygen saturation of 99% on room air. Physical examination was unremarkable, with a normal heart rate and rhythm and clear breath sounds.

**Figure 2:**
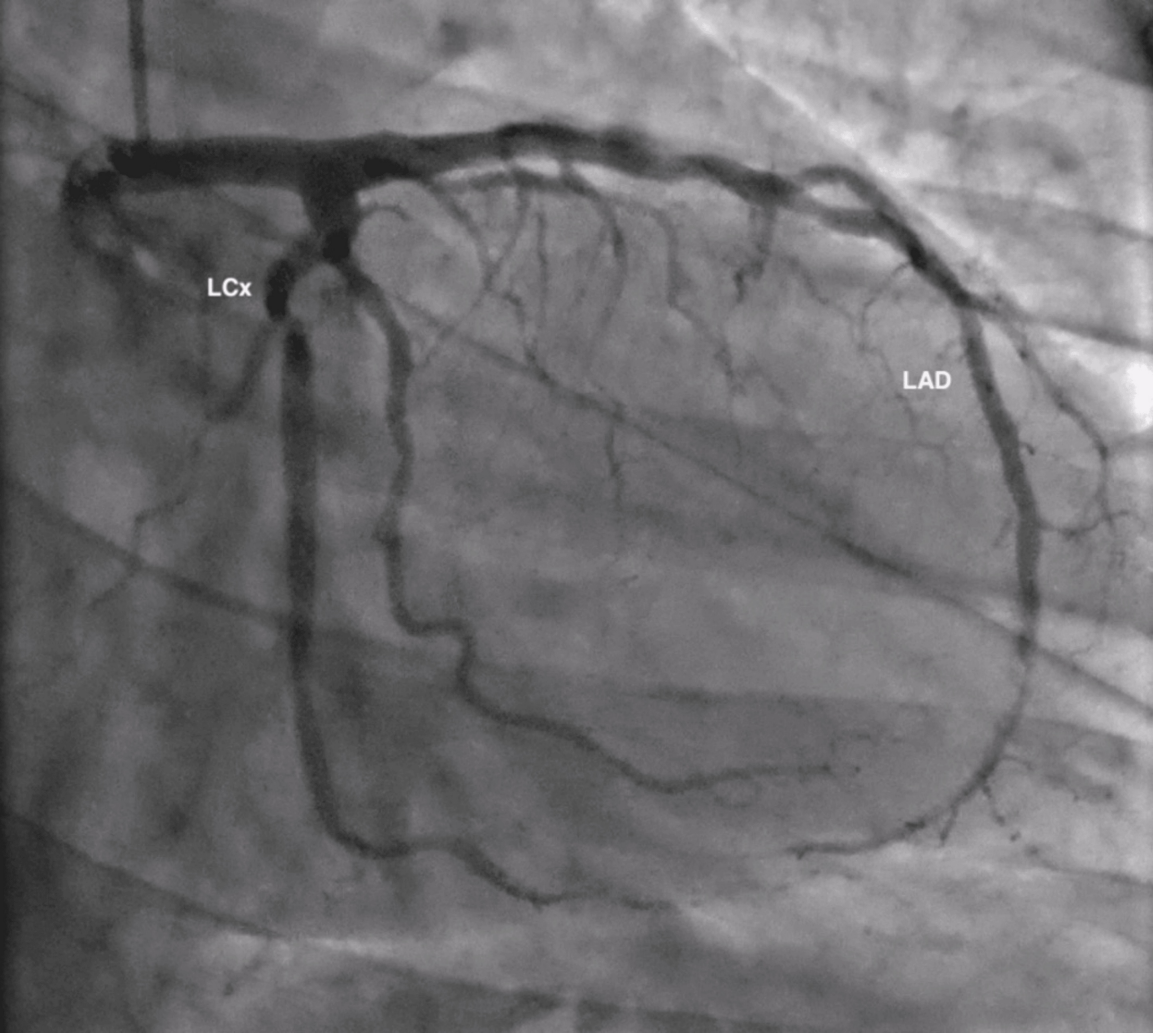
Left coronary angiography Demonstrating a 70% stenosis of the mid left circumflex artery (LCx), with mild diffuse disease of the left anterior descending artery (LAD) measuring approximately 30% stenosis and a focal 90% stenosis in the distal apical LAD.

The patient received thrombolytic therapy and was transferred urgently to our hospital for coronary angiography. During cardiac catheterization, the mid left circumflex artery (LCx) demonstrated a 70% hazy stenosis. This was treated via percutaneous coronary intervention and placement of a drug-eluting stent (Figure 2). Additional findings included mild diffuse disease of the proximal to distal left anterior descending artery (LAD) with approximately 30% stenosis and a focal 90% stenosis in the apical segment of the distal LAD. The RCA was not directly engaged during the procedure. Dual antiplatelet therapy with aspirin and ticagrelor was initiated.

**Figure 3:**
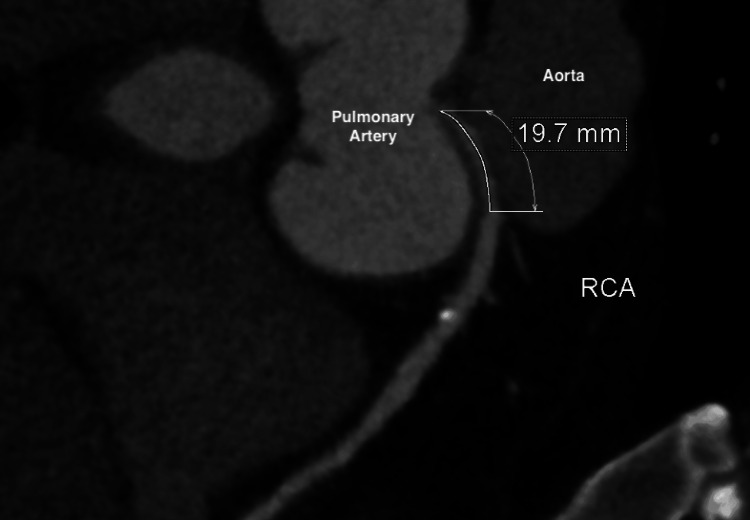
Coronary computed tomography angiography of anomalous right coronary artery Anomalous right coronary artery (ARCA) originating from the opposite sinus with an interarterial course traveling between the ascending aorta and the pulmonary artery. The proximal segment measures approximately 19.7 mm.

Subsequent coronary computed tomography angiography (CTA) was performed to delineate coronary anatomy further. CTA demonstrated an interarterial configuration of the ARCA, which traverses between the aorta and pulmonary artery (Figure 3). Given the associated risk with this anatomy, the patient was evaluated by cardiothoracic surgery for surgical management. Due to the recent STEMI and the need for uninterrupted dual antiplatelet therapy following drug-eluting stent placement, surgical intervention was deferred, and the patient was discharged with plans for elective surgical correction.

The patient was readmitted several weeks later and underwent coronary artery bypass grafting (CABG) with a graft of the left internal mammary artery (LIMA) to the LAD, surgical unroofing of the ARCA, and left atrial appendage ligation. The procedure was completed successfully without complications.

## Discussion

An RCA arising from the left coronary sinus with an interarterial course between the aorta and pulmonary artery is considered a malignant variant due to its association with exertional ischemia and adverse cardiac events [3,8]. Although the interarterial course identifies a high-risk anatomical configuration, contemporary evidence suggests that myocardial ischemia is more closely related to the presence of an intramural coronary segment within the aortic wall [2]. During periods of increased cardiac output, expansion of the aortic root may produce dynamic narrowing of this intramural segment. In addition, anomalous coronary arteries frequently demonstrate a slit-like ostium and acute angulation of the arterial takeoff, which can create a valve-like obstruction that intermittently limits coronary flow [2,3].

Although the acute presentation was attributed to obstructive disease of the LCx, the presence of a high-risk ARCA represents an independent substrate for ischemia and sudden cardiac death that would have remained unrecognized without advanced imaging. This case highlights the importance of evaluating for concomitant coronary anomalies when clinical presentation or angiographic findings are not fully explained by a single lesion.

Multimodality imaging is essential in identifying coronary artery anomalies. While invasive coronary angiography evaluates obstructive disease, coronary computed tomography angiography (CTA) provides superior visualization of coronary origins and their anatomical course [4]. Management options include RCA reimplantation to a more favorable position on the aorta, ostioplasty, pulmonary artery translocation, CABG, or unroofing of the intramural segment [2,4]. Unroofing is the most commonly performed repair and involves marsupialization of the shared aortic-coronary wall to convert the intramural tunnel into an open channel. Coronary reimplantation is preferred when the intramural segment is short or courses below the aortic valve, while ostioplasty may be added to address a slit-like ostium or acute takeoff angle. Pulmonary artery translocation, which involves anterior displacement of the pulmonary artery to relieve extrinsic compression on the interarterial segment, may be considered when the anomalous course is predominantly interarterial rather than intramural [4]. CABG is generally reserved for patients with concomitant atherosclerotic disease, as the intermittent nature of obstruction creates competitive flow that poses a risk for graft failure [2]. Early recognition and surgical correction may reduce the risk of ischemia and sudden cardiac death.

## Conclusions

This case emphasizes the importance of maintaining a broad differential when evaluating patients with acute coronary syndromes, particularly when the clinical presentation or angiographic findings do not fully align with a single culprit lesion. While the acute infarction was explained by the patient's LCx obstruction, the presence of a high-risk configuration of an ARCA represents an additional and clinically significant cause for ischemia that would have remained undetected without further evaluation. This case further highlights the value of multimodality imaging, as invasive angiography alone may not adequately define coronary origin or course, whereas coronary CTA provides critical anatomical detail that directly informs management decisions. Ultimately, early recognition of high-risk coronary anatomy allows for appropriate risk stratification, timely referral for surgical evaluation, and more comprehensive patient counseling, even when immediate intervention must be deferred, helping to reduce the risk of recurrent ischemia and adverse cardiac events.
